# Artificial Intelligence: Deciphering the Links between Psychiatric Disorders and Neurodegenerative Disease

**DOI:** 10.3390/brainsci13071055

**Published:** 2023-07-11

**Authors:** George B. Stefano, Pascal Büttiker, Simon Weissenberger, Tobias Esch, Maren M. Michaelsen, Martin Anders, Jiri Raboch, Radek Ptacek

**Affiliations:** 1 Department of Psychiatry, First Faculty of Medicine, Charles University and General University Hospital in Prague, Ke Karlovu 11, 120 00 Prague, Czech Republic; pascal_buettiker@hotmail.com (P.B.); sweissenberger11@gmail.com (S.W.); martin.anders@vfn.cz (M.A.); jiri.raboch@vfn.cz (J.R.); radek.ptacek@gmail.com (R.P.); 2 Department of Psychology, University of New York in Prague, Londýnská 41, 120 00 Vinohrady, Czech Republic; 3 Institute for Integrative Health Care and Health Promotion, School of Medicine, Alfred-Herrhausen-Straße 50, Witten/Herdecke University, 58455 Witten, Germany; tobias.esch@uni-wh.de (T.E.); maren.michaelsen@uni-wh.de (M.M.M.)

Artificial Intelligence (AI), which is the general term used to describe technology that simulates human cognition [1], has taken on a prominent role in twenty-first-century clinical practice and medical research. While many medical practitioners decry our increasing reliance on machines, AI has been particularly helpful for detecting and interpreting complex patterns in extremely large datasets that would otherwise go unrecognized. Recent advances include the development of machine learning (ML) algorithms that permit computers to learn from experience and adjust their output with no additional explicit instructions [2,3]. 

Despite its profound impact on other fields of medicine (notably, oncology and ophthalmology), AI has not yet been applied extensively to the screening, diagnosis, or pharmacological treatment of psychiatric disorders [4,5]. Psychiatric disease frequently emerges in adolescence and young adulthood with lifelong and unremitting symptoms that can have a devastating impact on quality of life. The World Health Organization estimates that one in eight persons worldwide is living with a psychiatric disorder [6], including nearly 300 million with depression alone [7]. While psychiatric disorders are currently diagnosed and categorized based on clinical findings as outlined in the Diagnostic and Statistical Manual of Mental Disorders (DSM)-V and the International Classification of Diseases (ICD)-10, categories and criteria change over time and are currently the subject of critical debate [8]. There are no imaging strategies or laboratory tests that can be used to diagnose these complex heterogeneous conditions nor are there any clear and objective criteria that predict treatment outcomes. Thus, while some individuals fall precisely into specific disease categories, many others exhibit signs and symptoms that are more complex and fluid. 

Given its capacity to evaluate and identify patterns in large datasets, AI is poised to make significant inroads in this field. Much recent interest has focused on natural language processing (NLP), which are algorithms that permit computers to understand and respond to spoken and written text. While introduced to the general public in 1968 in the futuristic fantasy film, 2001: A Space Odyssey, NLP is now part of our everyday lives (e.g., voice-activated devices and customer support interfaces) and holds great promise for the practice of medicine [9]. Several NLP programs have been developed to provide a form of online psychotherapy (e.g., Woebot, Sara, and Wyse, among others). These programs have been surprisingly successful for a variety of reasons [10], among them, increased access to care at a remarkably reduced cost. However, from the perspective of screening and diagnosis, researchers in this field are attempting to determine whether NLP can identify specific patterns of communication used by patients who have been (or might eventually be) diagnosed with mental illness [11,12]. Full application of this technology will require ongoing advances in NLP, including methods to streamline extremely large and somewhat noisy datasets. Likewise, many ethical and personal privacy issues will need to be addressed before, during, and after this methodology has been applied. Ultimately, NLP algorithms may also be capable of recognizing speech patterns in patients who have (or have not) responded to appropriate treatment and may be able to predict which treatment modalities might be most effective on an individual basis. Beyond NLP alone, AI and ML methods might also be used to identify objective biomarkers from complex metabolomic, genomic, and epigenetic datasets collected from large patient cohorts. These methods might also be used to assess subtle findings in electroencephalogram and brain imaging studies, including those that might not be apparent even to the most experienced neurologists and neuroradiologists [13,14].

Neurodegenerative diseases are disorders resulting from the death or deterioration of cells in the central nervous system. Many individuals who are ultimately diagnosed with neurodegenerative diseases present with psychiatric symptoms, including anxiety, major depression, and in some cases hallucinations and delusions [15,16,17]. While there are no known cures for these disorders or their psychiatric sequelae, several therapeutic strategies have been developed that can be used to manage them. For example, the atypical antipsychotic, pimavanserin, was approved in 2016 by the US Food and Drug Administration for the treatment of delusions and hallucinations, specifically in patients diagnosed with Parkinson’s disease psychosis [18].

In a recent publication, we highlighted AI applications developed for the early diagnosis of neurodegenerative disease [19]. This field was recently reviewed by Myszczynska et al. [20]. Among these recent applications, Yang et al. [21] published a groundbreaking AI study that identified nocturnal breathing disorders as an early objective biomarker of Parkinson’s disease (PD). Similarly, McKenzie et al. [22] used a deep-learning strategy to identify early histopathological and cognitive changes associated with Alzheimer’s disease (AD), and Bonnachi et al. [23] reviewed the use of AI to diagnose, predict outcomes, and monitor the treatment of multiple sclerosis (MS) based on magnetic resonance imaging findings and laboratory results.

As this field develops, we may soon be in a position to use AI to examine specific links between psychiatric disorders and progressive neurodegenerative disease [24]. While we already have a basic understanding of some of these similarities and differences, including features generally associated with neuroinflammation, serotonin signaling pathways, and the hypothalamus-pituitary axis [25], it would be extremely helpful to determine whether specific psychiatric symptoms (particularly those that develop in later life) might predict the diagnosis of one or more defined neurodegenerative diseases. Interestingly, results from a recent study of 500 elderly patients reported by Shdo et al. [26] revealed unique patterns of depressive symptoms (including dysphoria, hopelessness, withdrawal, worry, and cognitive problems) that predicted specific neurodegenerative diseases with 70–84% accuracy. Similarly, Stanton et al. [27] identified links between specific behavioral changes and distinct atrophic brain lesions. By contrast, results from an integrated genome-wide association/transcriptome/proteome study reported by Wingo et al. [28] highlighted a remarkable number of genetic and molecular similarities shared by neurodegenerative and psychiatric diseases. The ongoing integration of AI would permit studies such as these to be extended many-fold with more patient samples and further extrapolation from biochemical, genetic, and imaging data.

Many other critical questions might also be addressed by AI technology, notably those directed at predicting and monitoring responses to drug treatment and the selection of appropriate drug therapy for individual patients. AI may ultimately provide us with insight into the nature and treatment of psychiatric symptoms in all settings and may contribute concurrently to our understanding of neurodegenerative diseases. Of particular interest are findings that may lead to early identification of disease as well as new and effective prevention and treatment strategies.
